# From a decentralized clinical trial to a decentralized and clinical-trial-in-a-box platform: Towards patient-centric and equitable trials

**DOI:** 10.1017/cts.2023.629

**Published:** 2023-10-09

**Authors:** Dorothy Dulko, Manlik Kwong, Marisha E. Palm, Ludovic Trinquart, Harry P. Selker

**Affiliations:** 1 Institute for Clinical Research and Health Policy Studies, Tufts Medical Center, Boston, MA, USA; 2 Tufts Clinical and Translational Science Institute, Tufts University, Boston, MA, USA

**Keywords:** Decentralized, clinical trial, telehealth platform, recruitment, remote research data acquisition

## Abstract

**Background/Objective:**

Despite the intuitive attractiveness of bringing research to participants rather than making them come to central study sites, widespread decentralized enrollment has not been common in clinical trials.

**Methods:**

The need for clinical research in the context of the COVID-19 pandemic, along with innovations in technology, led us to use a decentralized trial approach in our Phase 2 COVID-19 trial. We used real-time acquisition and transmission of health-related data using home-based monitoring devices and mobile applications to assess outcomes. This approach not only avoids spreading COVID-19 but it also can support inclusion of participants in more diverse socioeconomic circumstances and in rural settings.

**Results:**

Our team developed and deployed a decentralized trial platform to support patient engagement and adverse event reporting. Clinicians, engineers, and informaticians on our research team developed a Clinical-Trial-in-a-Box tool to optimally collect and analyze data from multiple decentralized platforms.

**Conclusion:**

Applying the decentralized model in Long COVID, using digital health technology and personal devices integrated with our telehealth platform, we share the lessons learned from our work, along with challenges and future possibilities.

## Introduction

Historically, driven by operational convenience for the investigators, clinical trials have required research participants to travel to academic medical centers and/or community practices [1]. This centralized approach facilitates standardized processes and procedures that are important to the conduct of clinical research. However, the patient burden of cost and time associated with travel to visits is a barrier to trial participation, particularly for individuals living in low-income and rural areas [2,3]. Additionally, multi-morbidity and vulnerabilities related to age make travel to study sites and adherence to trial procedures difficult. In addition to restrictive selection criteria, the requirement to travel to sites contributes to highly-selected trial participant samples, and thereby lack of equitable clinical trials and fewer opportunities to examine heterogeneity in relative treatment outcomes [4].

Decentralized clinical trials are characterized by less dependence on traditional research facilities for data collection. In a fully decentralized trial, recruitment, delivery, administration of study medication, and collection of outcomes data all occur without in-person clinic visits between the study team and the trial participant. In a hybrid decentralized trial, participants may be on-site for certain study activities, particularly enrollment, and complete the rest of the scheduled activities at home [5]. A hybrid approach implements remote activities when feasible but also supports in-person activities as necessary, applying combinations of technologies with traditional in-person approaches. Determining the applicability of the hybrid design depends on the types of study assessments, procedures, safety considerations, and endpoints, e.g., drugs that require supervision administered in clinic, with follow-up decentralized telehealth visits. Decentralized trials present a way to enhance trial recruitment and retention by offering trials that are more flexible and less reliant on traditional research facilities [6].

There is a lack of standardized, shared terminology in the use of decentralized methods. Such trials have been labeled direct-to-participant trials, virtual trials, digital clinical trials, or remote clinical trials, which, paradoxically, emphasizes distance, causing confusion as decentralized trial activities are not necessarily “remote” from the participant’s perspective. Indeed, decentralized trials are centered around or closer to participants’ homes rather than at a traditional research site, distant from the patient [7]. A standard term, “decentralized clinical trials,” was recently used by the United States Food and Drug Administration (FDA) to describe trials in which patients participate at locations away from the investigator’s site [8].

Decentralized trials increasingly rely on digital tools employing technologies such as telemedicine, e-consent systems, mobile devices, wearable medical devices, and patient-driven virtual assessment interfaces to collect data [6]. Additional data collection can be achieved by using web-browser-based surveys (e.g., via REDCap), E-mail exchanges, and Short Message Service (SMS) text message-based surveys. Participant level of education and technical ability, the design and ease of use of the digital health technology, and whether the technology can be integrated with trial participants’ devices (e.g., their smartphone), are key considerations for investigators and clinical trial sponsors during development of decentralized design and methods [9].

Despite the intuitive attractiveness of “meeting patients where they are,” widespread adoption of a decentralized trial approach has not been achieved. Identified barriers include underdeveloped digital infrastructure, lack of experience with design, and concerns about regulatory requirements for data collection and reporting [5]. Another challenge is establishing a relationship between the study team and wearable device manufacturers, to obtain the quality and quantity of data needed for a particular study. Time and resources are needed to establish data access, data use agreements and Intellectual Property issues. As of 2020, there were an estimated 4.7 billion active internet users, more than half of the global population, with nearly 92% of these users were unique mobile internet users [10] Internet usage among US adults has steadily increased from 52% in 2000 to 90% in 2019 [11]. Despite these compelling usage data, certain aspects of decentralized trial delivery may not be feasible in all geographic locations. For example, in rural locations connectivity is not always reliable, consistent use of decentralized trial applications is not assured. The Federal Communications Commission estimates that more than 21 million people in the US do not have a reliable internet connection, including nearly 3 in 10 people (27 percent) who live in rural locations and 2 percent of those living in cities [12]. Other research, including analysis from Microsoft, suggests that there may be more than 160 million Americans without broadband or internet access with download speeds of at least 25 megabytes per second (Mbps) and upload speeds of at least 3 Mbps [12].

Beyond internet connectivity limitations, fully decentralized clinical research trials may not be feasible or desirable in certain conditions, such as in oncology trials where intravenous drug administrations, medical imaging, and complex adverse event assessments are integral to evaluating trial endpoints and patient safety. Yet for many other conditions, decentralizing certain study components may make traditional trials more efficient and patient-centric, reducing participant burden, thus enhancing trial retention and resource utilization [13]. Examples of chemotherapy and monoclonal antibody treatments administered in-home during the COVID-19 pandemic have the potential to broaden and modify the application of decentralized trials with participants being on-site for certain study activities while completing other activities from their home [14].

To our knowledge, although an increasing number of clinical trials integrate digital technologies [26][Bibr ref26], academic platforms to run decentralized trials are scant. The Eureka Research Platform, a digital platform funded by National Institutes of Health (NIH) and housed at University of California San Francisco, enables the design and execution of decentralized trials for researchers across the US. [27]. The Scripps Research Digital Trials Center partnered with CareEvolution, a health technology company that provides their MyDataHelps™ platform for decentralized trials [28]. Informed by Clinical Trials Transformation Initiative (CTTI)’s Digital Health Trials Recommendations, ETH Zurich created a Digital Trial Intervention Platform (dTIP) to design and conduct decentralized trials [29].

Our approach broadens the landscape of academic platforms for decentralized trials with unique “trial in a box” features. Recent institutional infrastructure investments at Tufts include an electronic Institutional Review Board (eIRB), Clinical Trial Management System (CTMS), and a Tufts Medicine-wide EHR, each supporting fully-integrated management and real-time monitoring of clinical trials, including tracking Investigational Products and trial- specific technology (e.g., wearables) shipped directly to study participants.

Our decentralized trial platform integration manifested through a transformation of our Informatics Program over the past five years, with expanded leadership and combined with staff and major institutional infrastructure investments. In the last few years, our team has developed and deployed a decentralized clinical trial platform, the Tufts Remote Support Platform and Engagement for Clinical Trials (R-SPECT), to support patient engagement, adverse event reporting and sample collection during the COVID-19 pandemic. Working with our engineering colleagues to develop a *Clinical-Trial-in-a-Box* tool, and with informatics collaborators we considered how best to collect and analyze data from multiple decentralized platforms while supporting participant retention, study integrity and sample collection by leveraging telehealth sample collection observation. The Tufts R-SPECT platform includes, however is not limited to, a physical *Clinical Trial in a Box*. This “trial agnostic” box prototype enables standardized delivery and inventory of study materials provided to enrolled participants, including remote wearable devices and other trial-specific supplies that are easily customizable to specific study requirements and endpoint measures. The Tufts R-SPECT platform includes remote patient enrollment, encrypted document generation, and integration of HIPPA-compliant telemedicine platforms for e-consenting and collection of e-patient–reported outcomes. The confluence of our expanded health-related data resources and new methodological approaches and data analytics capabilities are integral to R-SPECT. The Tufts Clinical and Translational Science Institute (CTSI) Health Informatics Program is an active collaborator in development of an effective digital ecosystem ensuring data security and interoperability. In this manuscript, we share the lessons learned from this work, including the challenges and future possibilities.

## The COVID-19 Pandemic and Decentralized Clinical Trials

The COVID-19 pandemic significantly disrupted clinical care and research across the US and the world [6]. Although telehealth technology and home health care were established in clinical practice prior to the pandemic, COVID-19 forced rapid-cycle adoption of crisis-related policies and procedures establishing pandemic standards as the new normal [13]. Organizations had to be nimble in adopting alternatives to the traditional top-down approach to clinical trials, with emphasis shifting to avoiding having clinical trial participants come to healthcare facilities when their care did not require it and to accommodate research teams working remotely [15]. Virtual interactions between clinicians and patients to provide continuity of care while maintaining social distancing, coupled with improving smartphone health applications and connectable medical devices, hastened the shift to decentralized trial models. The COVID-19 pandemic was a call to action to expand the use of decentralized and virtual interfaces while testing the feasibility of hybrid decentralized trial models incorporating traditional in-person and virtual modalities, fostering wider and more generalizable research [16].

Early in the COVID-19 pandemic, a collaboration between Tufts Medical Center and Tufts University brought together a multidisciplinary team to conduct an NIH National Center for Advancing Translational Sciences (NCATS)-supported decentralized clinical trial. The study recruited outpatients with mild to moderate COVID-19 to determine whether a widely available repurposed therapeutic, niclosamide, reduced SARS-CoV-2 oropharyngeal (OP) and fecal shedding and duration of COVID-19 symptoms [17]. Beyond testing the efficacy of the drug, overarching goals in the conduct of this decentralized trial included: (1) avoiding having patients visit a healthcare facility enhancing convienence of participation,  (2) reducing outpatient visits to limit infectious exposure of other patients and healthcare providers, (3) providing extensive decentralized patient screening prior to enrollment to ensure that all enrolled persons met inclusion and exclusion criteria (including avoidance of enrolling persons already severely ill with COVID infection), and (4) conducting in-home participant monitoring post enrollment via a Health Insurance Portability and Accountability Act of 1996 (HIPPA)-compliant telehealth platform to evaluate for progression to severe COVID.

## Decentralized Recruitment

Participants were identified from records of outpatients who came to Tufts Medical Center seeking COVID-19 testing. The Tufts Medical Center Institutional Review Board (IRB) approved an omnibus information sheet, in multiple languages, for display at the Tufts Medical Center outpatient laboratory stating that patients requesting COVID-19 testing might be contacted by a research study team. Each Tufts Medical Center and affiliate site displayed a study-specific poster with QR code directing potentially COVID positive patients to study team contact information in the emergency department (ED), primary care, and infectious disease clinic of each Tufts site. In additon, any patient with mild to moderate COVID-19 symptoms presenting to the EDs or clinics who did not require supplemental oxygen or hospitalization received a brief overview of the study from the local clinical team with contact information for the study team at the time of testing. Patients were advised that following confirmation of a positive COVID-19 test they could proactively engage with the study team if interested in participation.

During trial activation, we simultaneously built a corresponding Research Electronic Data Capture (REDCap) database as this electronic-database capture was readily available within the Tufts CTSI infrastructure. A structured data query and a secure automated data delivery process were developed by the Tufts CTSI Informatics Team that created a data file of outpatients testing positive for SARs-CoV-2. These data were electronically provided to the study team twice daily, at 7am and 3pm, via REDCap. The data files were housed in a secure directory with access limited to members of the study team, password protected. The Tufts CTSI Central Screener quickly identified and broadly screened each potential participant positive for SARS-Cov-2, referring them directly to the study team through an automated e-mail notification in REDCap.

## A Successfully Deployed Decentralized Clinical Trial

**Figure 1.**
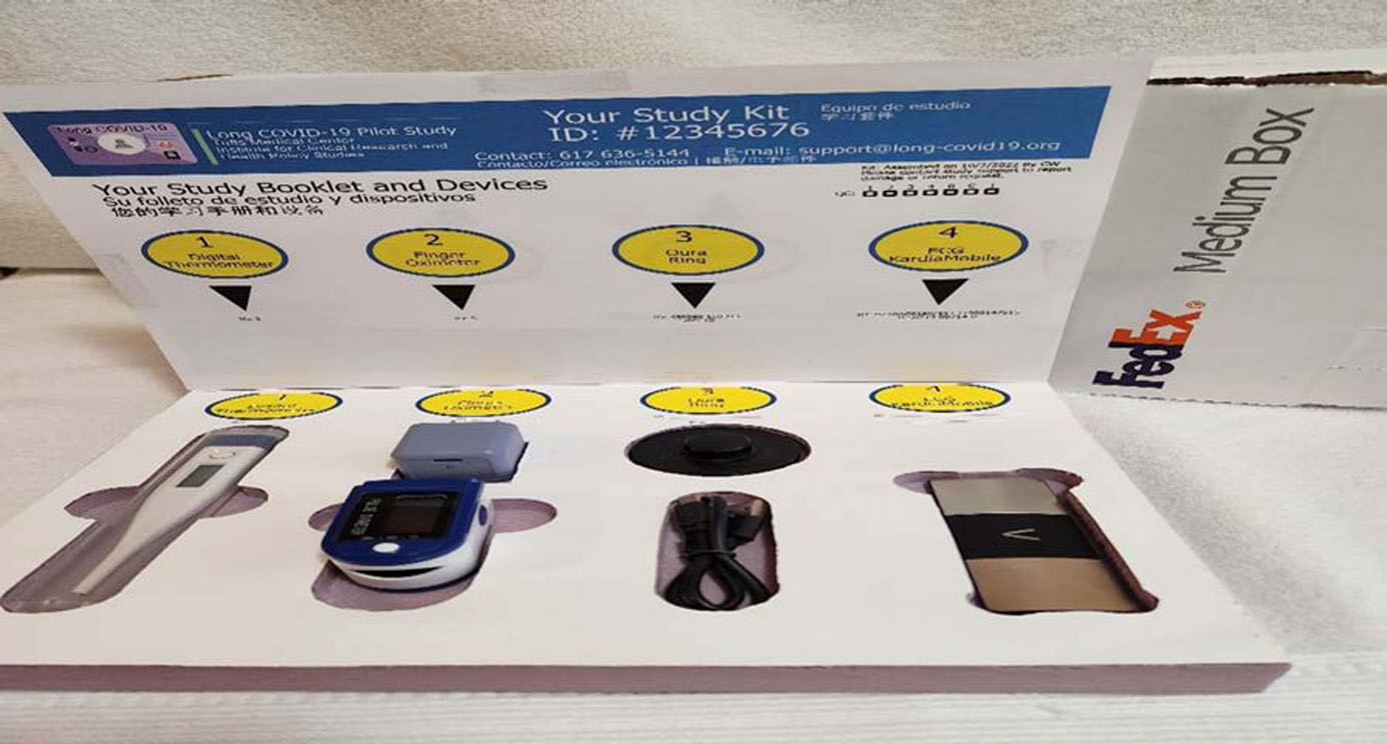
Decentralized clinical trial box prototype.

Upon enrollment and consent, each study participant received a *Clinical-Trial-in-a-Box* – including a thermometer, an oximeter, and oropharyngeal and fecal sampling materials with materials to return self-collected specimens, delivered directly to their home (Fig. 1). The boxes were barcoded, scanned, and delivered by a HIPPA and Occupational Safety and Health Administration (OSHA)-trained and certified medical courier. Strict chain of custody protocols from the moment of pick up to drop off were maintained by the courier service. Our Tufts R-SPECT platform was used to conduct all study visits including e-consenting, monitoring of symptoms, assessment of adverse events, and coordination of participant self-collection and return of oropharyngeal and fecal specimens. The blinded study drug (or placebo) was dispensed, sealed, and labeled by the Tufts Research Pharmacist with direct hand off to the study coordinator, maintaining Investigational Drug Accountability Record Forms. The study coordinator retrieved the investigational product from the research pharmacy for inclusion in the study box. A certified partner courier (HIPAA compliant and OSHA compliant) was notified of a ready pickup and received the study box via direct hand off from the study coordinator for immediate delivery to each participant. A proof of delivery photo was provided in real-time to the study team by the courier.

The trial opened to accrual on October 1, 2020, and the last participant was enrolled on April 20, 2021. In all, 73 participants were enrolled and randomized [17]. The study team completed 728 telehealth visits with participants. Over 650 SARS-CoV-2 test samples were self-collected by study participants: 316 respiratory samples and 348 fecal samples.

**Figure 2.**
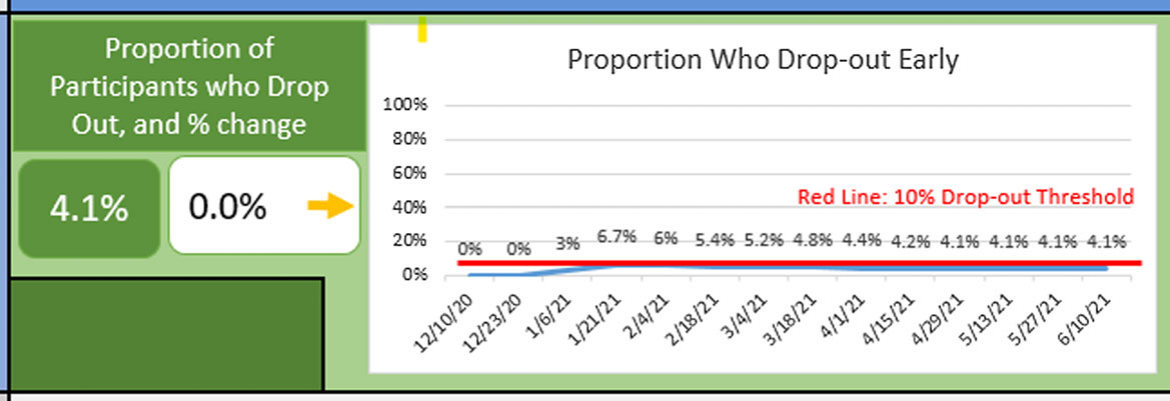
Proportion of patients who droped out prior to end of study.

Of the 67 eligible trial participants invited to take part in a post-study mixed methods evaluation of the telehealth platform, 46% (n = 31) completed a post-trial survey to determine participant and research team perspectives of the decentralized trial [16]. Key findings included:

- Compliance with planned telehealth visits ranged from 84% to 100% over the course of the study.
- Fecal and respiratory (oropharyngeal) samples were self-collected with a mean of 94.6% of planned fecal specimens and 96% of respiratory specimens successfully collected and processed across the duration of the study.
- The proportion of participants who dropped out of the study early was sustained below the target threshold of 10% (Fig. 2).
- Ninety-seven percent (*n* = 30) of post-trial survey respondents had not previously participated in clinical research; 77% (*n* = 24) of whom said they would consider taking part in another clinical research study in the future for a condition other than COVID-19.
- Of those who reported they would consider participating in a future study, 96% (23/24) indicated they would be more likely to participate if the visits were not in-person.
- The greatest barrier identified (*n* = 3) involved technology issues with the telehealth visits, including poor internet connection or cell phone coverage. However, those who identified this challenge said when one platform was not working, the researcher and participant would switch to a second platform, causing minimal disruption.

We recognize that the 46% of participants who completed the post-trial survey are a subset of all participants and there may be response bias. However, this information does provide some useful data regarding the feasibility of remote patient self-collection of specimens.

Among all patients in the Intent-to-Treat sample, there were 4 African American individuals (6.0%), 5 Asian individuals (7.5%), 7 Hispanic individuals (10.4%), 53 White individuals (79.1%), 1 individual with multiracial or multiethnic background (self-reported Asian and White; 1.5%), and 4 individuals with other race or ethnicity (6.0%). All participants were from Massachusetts. While state-to-state licensing accommodation was an option during the pandemic, we did not need to identify physicians with medical licenses in multiple states.

The team acknowledges the relative lack of ethnic diversity in our trial and that input from diverse participants is paramount to ensuring that trials are responsive to all patient needs while also meeting research requirements including gaining ethnically diverse perspectives of interfaces and data collection tools. Our future work includes collaboration with the Tufts CTSI Community and Stakeholder Engagement team to design studies that foster gaining this important perspective. In the next phase of development, we will engage the Tufts CTSI Stakeholder Expert Panel, a group of local community members, in providing feedback on the R-SPECT platform and its processes in order to ensure that it meets the needs of diverse patients across the geographic spectrum. With the median age of 31 years in our initial COVID-19 decentralized trial, our study demographic may indicate that younger people were more comfortable with decentralized technology, telehealth visits, or self-sampling. Further study is needed with older adults as well as with special populations [16].

## An Academic-Engineering-Manufacturing Collaboration

While the Tufts R-SPECT platform was first deployed during the pandemic, we believe it has broader applicability for the conduct of clinical research. Collaborating with the Massachusetts Institute of Technology (MIT) Master of Engineering in Manufacturing Program, along with clinicians and researchers at Maine Medical Center, we are engineering and testing our decentralized clinical trial platform with enhanced workflow processes and a more user-friendly interface for use in a range of settings. Key considerations informing platform design include: (1) interfacing with wearables, smartphones, and other devices; (2) capturing electronic patient-reported outcomes; (3) CTMS integration; (4) supporting use of diverse learning products (print, video, animations, and images); and (5) managing and tracking study inventory.

Our v2.0 *Clinical-Trial-in-a-Box* contains home-delivered trial participation materials, including consumer wearables with data access agreements and adaptable learning products (print, video, animations, and images). Logistics and tracking managing inventory not shipped, items shipped, delivery, and replacement management are integrated within the CTMS. Through this collaboration, the platform became more capable of outreach for decentralized trials to populations of patients that often do not participate in clinical trials, e.g., rural communities where a clinical visit can be an hour or more away.

The overarching objectives for the Tufts CTSI-MIT ongoing collaboration are to: (1) develop a platform to run a fully decentralized trial from enrollment to participation, (2) integrate reporting systems and metrics at the study level, (3) customize home delivery of trial materials, and (4) collect patient-reported and Social Determinants of Health (SDoH) data. This last goal was a primary objective, as including participants with a range of SDoH is key to gaining a deeper understanding associated with barriers to trial participation, specifically in under-resourced populations. Based on our initial work, patients who may have faced challenges to involvement in centralized trials appear to be able and willing to consider enrollment in a decentralized trial. We also seek to foster enhanced trial access for other populations currently underrepresented in traditional trials, e.g., the elderly, those living in remote locations, and racially and ethnically minoritized groups.

## Decentralized Clinical Trial Data Acquisition

**Figure 3.**
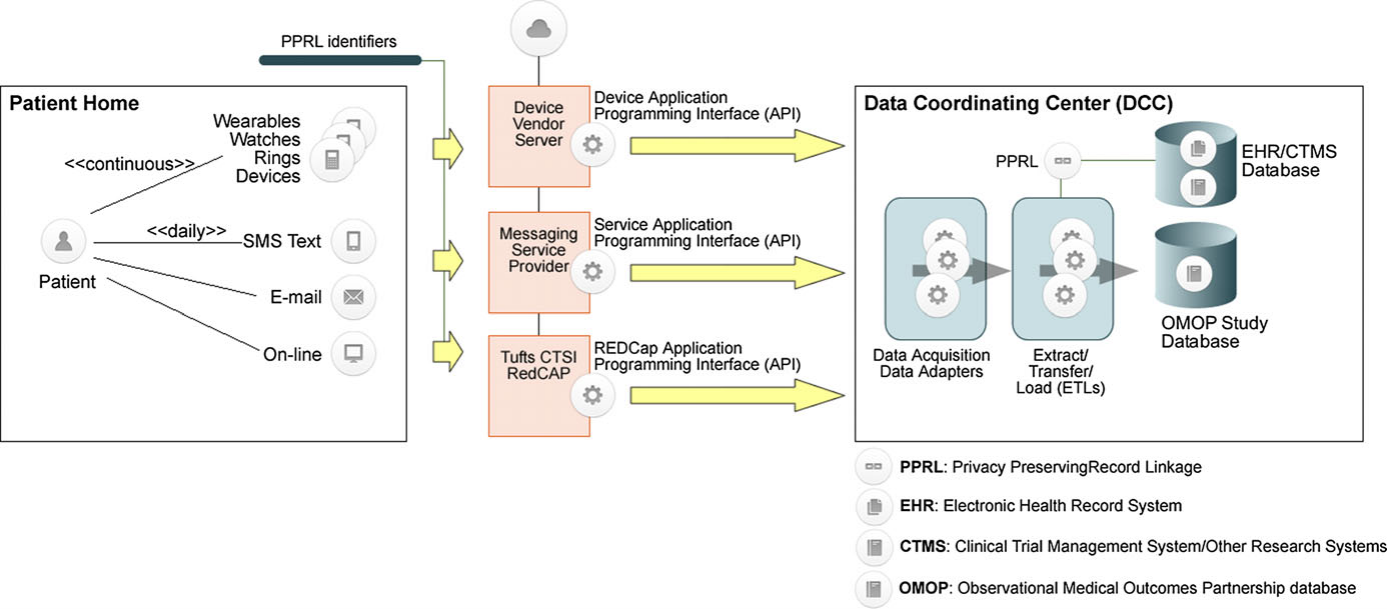
Decentralized clinical trial platform data acquisition process/systems.

The privacy of data acquisition is a key consideration, as is blinding, as investigators or sponsors should not see certain data. Digital health technology must ensure privacy and security to prevent unauthorized access to the technology and the data it collects [8]. Privacy-Preserving Record Linkage (PPRL) enables the de-identified linking of individual-level data across time and data sources to mitigate privacy concerns through encryption of personally identifiable information [18]. In traditional clinical trials in which data is collected onsite by investigators and research study staff, there often is no need for PPRL. In a decentralized-based platform, particularly where home and consumer-grade medical devices (i.e. smart watches, smart scales, smart thermometers, electrocardiographic devices such as the KardiaMobile 6L, etc.) are used to acquire trial measures, data may first be uploaded to the medical/consumer device cloud server under different user identifiers which often have the same data elements as are captured within a traditional clinical trial; e-mail address, first/last name, date of birth, and addresses. The externally staged data need to be linked via PPRL methods to the core patient medical histories and data from the study “home” research site or clinical information system. In a decentralized clinical trial platform, we do not make the assumption that all data involved are easily and readily accessible. In its design and implementation, we envision providing tools and services to support PPRL when needed. The data acquired from disparate devices, systems, and methods produce vendor proprietary data formats that can be normalized and aggregated into a central data collection center, e.g., at the Tufts Research Data Warehouse, for management, quality assessment, and analysis (Fig. 3).

In our ongoing Long-COVID study, decentralized trial participants are assigned study-specific e-mail addresses that provide a level of identity protection and enable linkage to other study systems such as previous care data from the EHR or within a CTMS. With assigned e-mail and SMS Text messaging, study participant identity is protected when handling responses to patient-reported surveys, a component of our PPRL approach.

The assignment of study-focused e-mail addresses has been applied in a number of MIT-run studies involving Oura® rings and other wearable devices. Using study e-mails anonymizes patients’ participation and data in terms of its relationship with wearable device vendors and data acquisition/staging solutions. We do not expect study participants to actually use these study-assigned e-mails. However, there could be a protocol in which the study participants might use the assigned e-mails to access wearable device portals and resources that are completely independent of their personal e-mail accounts. Building on previous work, global unique identifiers are generated for each study participant using a hash function on the data source identifier and other known descriptors of the study participant [19].

A shared Common Data Model (CDM) and standardized language are capabilities critical to the overall successful execution of decentralized studies. As such, Tufts CTSI has implemented the Observational Health Science and Informatics (OHDSI) Common Data Model to represent EHR patient care and outcomes data. The Observational Medical Outcomes Partnership (OMOP) is a public-private partnership, chaired by the FDA, administered by the Foundation for the National Institutes of Health, and funded by a consortium of pharmaceutical corporations in collaboration with academic and clinical researchers. The OMOP established a research program seeking to advance the science of therapeutics’ safety surveillance applying observational healthcare data [20]. The OMOP experiments demonstrated feasibility of establishing a common data model and standardized vocabularies that could accommodate different data types from various care settings represented by different source vocabularies in a manner that could facilitate cross-institutional collaboration and computationally efficient analytics. This supports the ability of connected systems used in the conduct of clinical research to effectively and securely exchange information, integrated into the data platform. This concept is important since many digital device technologies are developed by consumer health care companies and may not be intended to interface with a clinical data collection tool. The OMOP provides a standardized format that fosters patient data collection in a single space, facilitating accessibility and analysis.

To further enable data integration from decentralized trials to existing patient care, data are mapped to standard OMOP concepts and transfered to an OMOP study database. Then SMS text messaging is used to send and retrieve survey data, with survey questions and responses mapped to standard concepts, managed within an OMOP study database. Having study data encoded and managed within an OMOP database leverages standard tools and support from the international community of OHDSI investigators. Recognizing the technical challenges of conducting research across disparate observational databases, the Tufts team adopted the OMOP Common Data Model as a mechanism to standardize the structure, content, and semantics of observational data, making it possible to input statistical analysis code that could be readily adopted and re-used at every data site [21]. In our experience, the generalized and patient-centered design of the OMOP Common Data Model makes it a viable database schema to manage clinical trial data. The underlying value proposition is that OMOP Common Data Model supports standardizing data at study initiation, saving time and effort at study closure where data must be cleaned, normalized, etc., allowing for formation of study tracking, reporting, and dissemination based on established standards.

## Building Blocks for a Decentralized Platform

**Figure 4.**
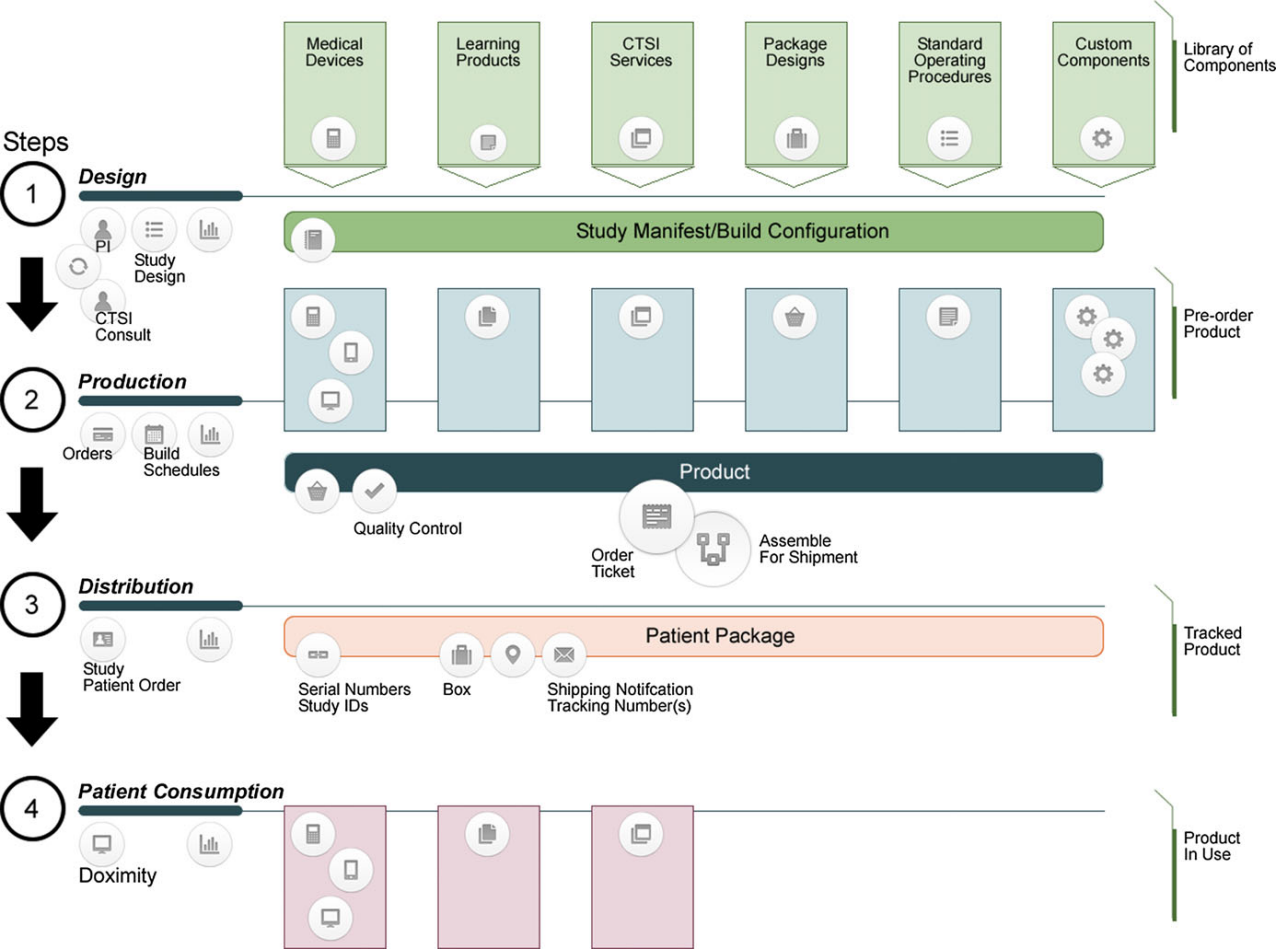
Decentralized clinical trial manufacturing process.

With each unique decentralized trial applying digital health technology, a catalog of standards-based data acquisition solutions and relationships must be built with various vendors. Each trial-specific device, as well as in-hospital or clinic-based devices, is a Lego®-like piece requiring data mapping, patient-centered documentation, and a study deployment process. Each newly designed study can draw from this warehouse of Lego®-like pieces to build and customize a unique solution for conducting their specific trial. The use of the term Lego®-like is descriptive and intended to convey our approach to designing the software and process infrastructure as reusable components rather than “one-off” structures and solutions that are discarded at the end of each clinical trial. While every study will require different instruments and solutions, our informatics approach is based on decades of designing software and processes using Object Oriented Design/Programming (OOD/P) principles and Design Patterns. We are applying those well-tested principles from software development to the process of clinical trial platform construction. The Tufts CTSI Decentralized Trial Team provides researchers consultation, production assembly of components, and database building support for this work. In the ongoing Tufts CTSI-MIT, Long-COVID collaboration, engineering students work in small teams to assess and develop decentralized clinical trial components such as packaging, distribution, inventory management, and other components that continually evolving digital health technology specifics, patient-centric solutions, and logistics in accounting of and managing devices and processes (Fig. 4).

## Ongoing Decentralized Trial Work at Tufts

Building on the successful implementation of our Tufts R-SPECT platform during the pandemic and our Long-COVID work, we are expanding the reach of an enhanced platform as a demonstration project within an N-of-1 trial design, first focusing on the chronic conditions of rheumatoid arthritis and asthma. N-of-1 trials are single-patient trials in which study participants are given FDA- approved treatments in a randomized order and a double-blinded fashion. Structured data collection is applied to determine the most effective treatment for that individual. N-of-1 trials are particularly amenable to our R-SPECT platform which enables seamless integration with consumer health devices, bi-weekly telehealth interaction with patients, collection of patient-reported data via mobile platforms and onsite structured clinical assessments. Selker and colleagues published “A Useful and Sustainable Role for N-of-1 Trials in the Healthcare Ecosystem” which presented a comprehensive review of opportunities and approaches for N-of-1 trials [22]. In this review, we acknowledge that standardized creation of N-of-1 trial software platforms and services has sustainability challenges. For rare and ultrarare diseases, and for precision treatments based on individual patient characteristics and responses, N-of-1 trials may provide a paradigm for the collection of data, homogenizing N-of-1 protocols and decentralized platforms applied within geographically diverse sites. While it is well known that the impact of decentralized trial methods varies, we propose that in conditions where the patient population is small and visits to a central research site may be very costly and burdensome to participants, there is an opportunity to evaluate further opportunities and challenges. Our research collaborators at the Tufts Center for the Study of Drug Development (CSDD) are evaluating the financial aspects of N-of-1 trial by conducting a longitudinal study with specific decentralized trial approaches and technologies, facilitating analysis by participant demographics, trial phase, trial complexity, and other key trial operations metrics [30].

We anticipate facilitating clinician engagement and patient participation by reducing the number of in-person visits via an adaptive *“Clinical-Trial-in-a-Box.”* This will allow integration of condition-specific electronic Patient-Reported Outcomes collection. To support participants in rural settings, we plan to collaborate with pharmacy chains that perform relevant laboratory tests. For participants without internet service, decentralized collection of self-reported data could be handled by Web-enabled platforms at local pharmacies and community centers.

We will use SMS text messaging to send and retrieve survey data, and questions and answers will be mapped to standard concepts and managed within an OMOP study database, as done now in our Tufts Splenic Tumor Assessment Tools (T-STAT) and Long-COVID-19 study. Having study data encoded and managed within an OMOP database takes advantage of standard tools and support from the international community of OHDSI investigators. As an open-science standard, it is also sharable and can be aggregated with other OMOP implementations and institutions.

## Challenges to Decentralized Trials

As decentralized trials often employ wearable digital device technology, participants have different user interfaces. Lack of consistency across applications in terms of icons, text, fonts, color, and reactions to user actions, challenges seamless user experiences. As a consequence, requests for user support increase the need for responding study personnel who require training on various devices and applications. Although having only two predominant mobile phone operating systems (Android and Apple) reduces user experience variability to some extent, the service organization still must train research staff and have operational skills in multiple wearable devices and resources available to support the delivery of a decentralized trial, increasing trial costs.

In addition to user experience variability, the process of acquiring digital technology data is handled on an individual vendor basis, as there are no standards covering data integration and dissemination from wearable devices. AliveCor, for example, provides healthcare organizations with a central portal (KardiaPro) to manage all patients using their KardiaMobile devices to acquire and analyze ECG recordings. Other wearables such as Oura® and Garmin® have taken more highly customized approaches to data access and use agreements in enabling research teams to acquire patient data from their cloud systems. However, at present, none of these solutions subscribe to a common standard to represent the data. Open Authorization (OAuth) 2.0 is a common and modern standard for accessing resources from a third party and is being used to authenticate and execute daily downloads from AliveCor within our Tufts CTSI Long COVID-19 study. However, in our experience, not all third-party collaborators may wish to use this method for a particular study. For example, Oura® is another third-party wearable collaborator who currently prefers to use a secure batch data transfer method. It is important to have an infrastructure design that can accommodate and adapt to current and emerging standards.

## Opportunities and Future Direction

Development and innovation in technology accompanied by the disruption brought on by the COVID-19 pandemic, has resulted in further evolution of clinical trial conduct post-pandemic. The structures and processes for delivering decentralized clinical trials are not yet flawless, but there is a growing consensus that this approach may help to support data sharing and collaboration across settings. The real-time acquisition and transmission of health-related data using home-based, monitoring devices and mobile applications to assess vital signs, symptoms, adverse events, and other research outcome measures are key drivers of future decentralized trial research.

Decentralized clinical trials may allow recruitment and retention of participants regardless of where they reside or work and offer a solution to the cost and time burden of traveling to trial sites for multiple study visits. Because of this flexibility, decentralized trials may foster diversity in enrollment. Evidence continues to evolve assessing exactly how decentralized clinical trials perform relative to otherwise similar clinical trials that didn’t apply decentralized methods.

Social Determinants of Health, including socioeconomic status, race, and ethnicity create barriers to basic medical care and prevent minoritized groups from participating in clinical trials- decentralized or other. As individuals from racial and ethnic minority groups as well as rural populations may disproportionately live in areas without clinical research facilities, decentralized trials may open access to research.

The use of wearables, such as activity trackers, may herald more inclusive participation if made available to a diverse population. Decentralized clinical trials may reduce costs associated with recruitment and retention compared to conventional in-person trial designs and reduce costs for participants as well. However, because of digital divides, there is a risk that decentralized trials actually lead to less diversity and poorer representation of the individuals with the target condition. Individuals who are Black and Hispanic, older, have lower educational attainment, have lower household income, and live in rural areas are least likely to use the internet or have broadband internet access [31]. Despite this, some recent decentralized trials have had success in increasing participation of minoritized groups [25]. Given the importance of community engagement in decentralized clinical trials, we plan to apply our increased understanding of community-specific health needs and local structural and SDoH to promote increased study participant diversity and health disparities research. Integration of SDoH and community engagement will be key to our understanding of the impact that decentralized trial implementation could have on recruitment and retention, particularly of less often studied populations [23–25].

We acknowledge the small sample size of our decentralized niclosamide trial and this experience does not allow us to understand the range of challenges within the conduct of decentralized clinical trials. We continue to develop and implement our decentralized clinical trial platform as integral to our Long- COVID-study. Two additional remote trial platform pilot studies are currently underway at Tufts, a veterinary Tufts Splenic Tumor Assessment Tools (T-STAT) [32] in which SMS Text-based clinician and client remote outcomes survey is used and a pilot collaboration with MIT Lincoln Labs testing usability and data acquisition of Long COVID-19-related physiological parameters. Our work with N-of-1 trials presents an opportunity to evaluate a hybrid model to assess patient-reported symptoms and health-related outcomes to guide patient-centric management of chronic conditions employing the Tufts R-SPECT platform.
